# The Use of the Mallet

**Published:** 1866-11

**Authors:** Lucy B. Hobbs

**Affiliations:** Chicago, Ills.


### THE USE OF THE MALLET.

BY LUCY B. HOBBS, D. D. S., CHICAGO, ILLS.

[Read before the Iowa State Dental Society.)

ALTHOUGH among the later professions, yet there is none, to my knowledge,that has made more rapid progress,or attained a higher degree of perfection than dentistry, in its various branches. The mechanical, for many years, occupied nearly, if not all the time, and talent of the gifted in the profession, each vieing with the others to see who should make the greatest change in the prevailing system, till we had almost as many different ways of inserting, or preparing artificial dentures as we had dentists. That department of course, brought out all the talent of the profession, so that they soon had it to such a degree of perfection, that any *ordinary* dentist could make teeth to excell even nature itself, as we were often informed. Not so with the operative part; They did not even seek a change; but each operator followed on the well beaten track of his illustrious predecessor, year after year. True, there were some honorable exceptions to this assertion; yet, with the mass, it was true, as far as I have been able to learn from the perusal of the dental literature within my reach. I hope I shall be pardoned by the profession, if I make any wrong assertions, and that they will attribute it to the right direction, ignorance, not a wish to prevaricate, for they will remember that all the knowledge I have been able to gain has been from books, till within the past year; and too many times they leave students more in the dark than they were before the perusal. It appears that each operator was afraid to make any decided change in the system, as Prof. S. remarked one day "that it required more moral courage than most men possesed, to advocate a theory against so many learned men," so each followed on in the old system of hand pressure in its various forms, till Dr. W. H. Atkinson, one of those inventive minds (one who possessed assurance enough of his own merit, to bring it before the profession) conceived the idea of the use of the mallet in condensing gold fillings. It was about the year 1858 or '59 that he first introduced it to the profession in the Convention; though, I believe he claims to have used it in practice some time previous. Prof. Taft, being one of those ready to grasp any new idea, if it had the slightest chance of success, soon commenced the use of it; and became a more earnest advocate than its founder, if possible. Thus without any controversy, and very little writing, the profession have unanimously adopted it. It is such a self-evident fact, that it is the best method, that one and all think it unnecessary to waste time to write about it; for I have been unable to find one article in the Journals pertaining to it. I am led to believe that this must be the reason why I was requested to write upon this subject to expose my ignorance. It was not long before many of the older operators, who had sacrificed their health, and almost their lives, in endeavoring to advance the profession, found that they could operate hours with less fatigue and do better work with less strength, than formerly; and thus, by degrees, from various reasons, it has become the prevailing system among the best operators. It needs but a few facts to show to every thinking mind, that it is the best system yet known to the profession for all ordinary fillings, as very few but can be better and more easily condensed than by hand pressure. No proof is necessary to show that any one can do better work, when he can give all his attention to the placing of the gold in the cavity, stand easy and natural, and have an assistant do the condensing. In the old way the operator was all worn out with a few fillings. The position was such that in most cases the strength could not be applied in the right direction, but at a great disadvantage to the operator, so that after a very few years of practice an operator was worn out ere he had arrived at any degree of perfection. Any one that has tried both systems, will admit that more gold can be condensed in a cavity, and, of course make better filling, as it is more solid, than in any other way, as instruments have fine serrated points, and as it is driven firmly to place by the mallet it leaves deep points to be filled, and, of course makes good receiving surface for the next block, and so on, till it is one solid mass. Let the gold be used in any form you choose, it can be better condensed with the mallet than with the hand. To make good fillings with the mallet, one must have good, deep retaining points. In some instances, it is very difficult, if not impossible, to make a good filling with the mallet, in the usual way, for instance, in broad shallow cavities, where it is impossible to get retaining points, the gold will be quite likely to roll out in one solid mass, to the great annoyance of the patient, and extreme mortification of the operator, The humane operator will give it a fair trial. For there are none but are well aware that it requires strong nerves to endure the hand pressure, without some manifestation of suffering, but with the mallet, I have known the patient to sleep, when the operation was long, thus showing that it was not very unpleasant. Some claim that it is beneficial in cases of periostitis ; but how the cure is effected, or what its *modus operandi* is I am unable to say. One great benefit which the profession will derive from it, is the superior advantage of the young members to learn operative dentistry. Formerly the visits to the operating room were few and far between. Patients objected to their presence; but now it is necessary. In condensing with the mallet little instruction can be given, a few hints are all that are necessary to the most unexperienced. Let the operator hold the instrument in such a manner that every blow will drive the mass to the walls of the cavity, so that a fine solid margin is made to the filling, thus protecting the edges of the enamel, from the fluids of the mouth. Some times the operator is much annoyed by the gold clinging to the instrument, (especially if the serates are deep) and pulling away the blocks previously inserted. To avoid this, the best method I know of is to remove the pressure which all operators give, I believe as they place each piece of gold in position, and as most of them condense with two blows of the mallet, the first driving the gold firmly into the block previously placed, as the second blow, in being struck, removes the pressure on the instrument, and by applying just force enough to hold it in position, the second blow will condense just as much as the first, and at the same time loosen the instrument from the gold, so that there will be no tearing away of the parts, this is more necessary in the use of the pellets of adhesive gold, than in the block or cylinders, which are less liable to displacement. In condensing, it is better not to pass the instrument closely over all of the part of the gold previously inserted, for it becomes so cut up and hardened on the surface that the gold will not adhere, but clean away in pieces, thus making it almost impossible to build up a tooth properly. Feeling that what little I may be able to say upon the subject is so much better understood by the profession than I can possibly be expected to understand it, I feel like asking pardon for attempting it, and consuming so much valuable time and not rendering a just equivalent for it.

